# Supplementary material for: The secreted protease Adamts18 links hormone action to activation of the mammary stem cell niche
Source: Nat Commun. 2020 Mar 26;11:1571. doi: 10.1038/s41467-020-15357-y (PMC7099066; doi:10.1038/s41467-020-15357-y)
Supplement: Supplementary file 1 — Supplementary Information [file 41467_2020_15357_MOESM1_ESM.pdf]

Supplementary Information

The secreted protease Adamts18 links hormone action to activation of the mammary stem cell niche

Dalya Ataca, Patrick Aouad, Céline Constantin, Csaba Laszlo, Manfred Beleut, Marie Shamseddin, Renuga Rajaram, Rachel Jeitziner, Timothy Mead, Marian Caikovski, Philipp Bucher, Giovanna Ambrosini, Suneel Apte, Cathrin Brisken

Supplementary Figure 1

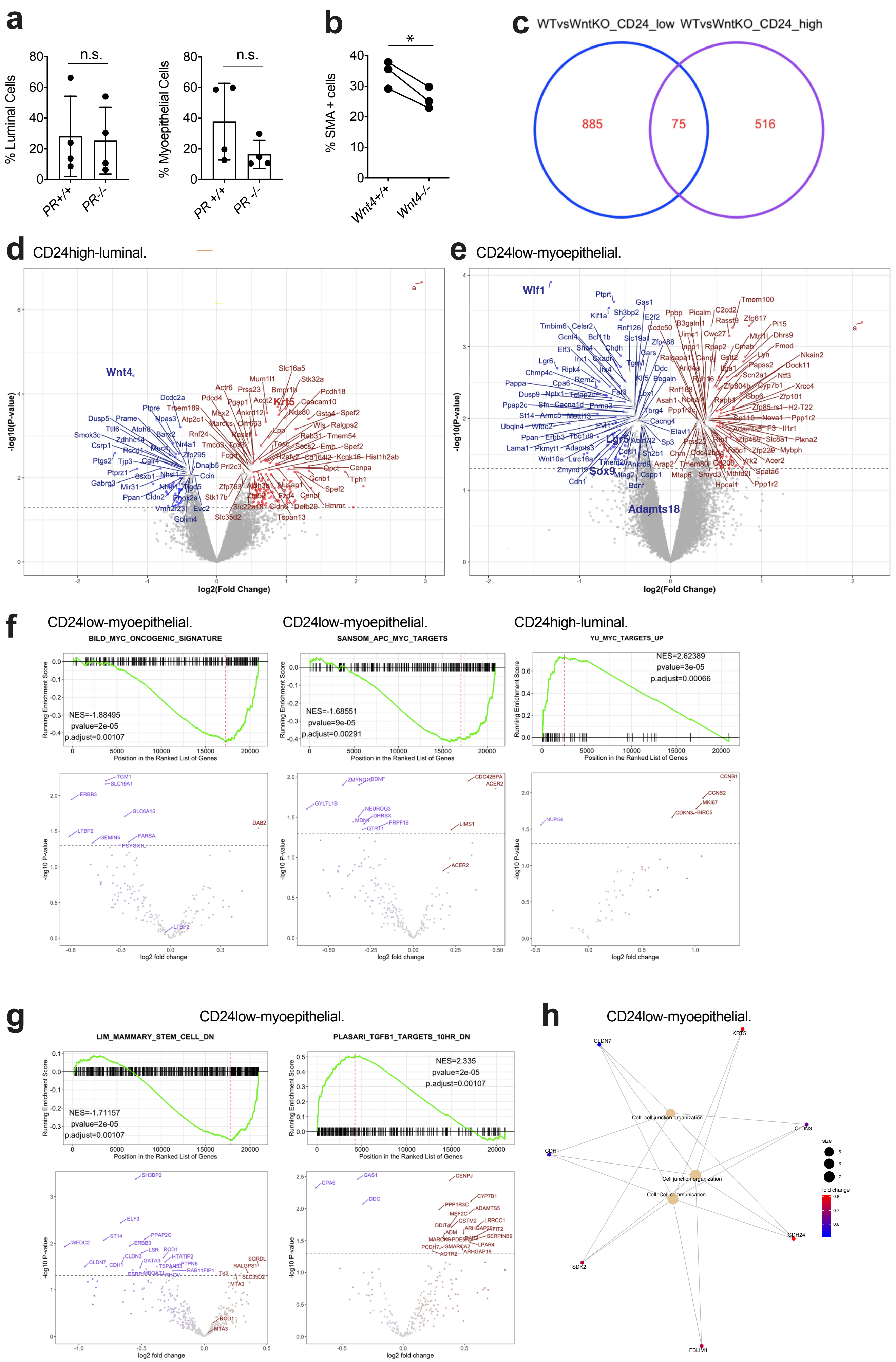

Supplementary Figure 1. Transcriptomics of *Wnt4*$^{-/-}$ mammary epithelial cells

a Bar graphs showing percentage of luminal and myoepithelial cells among the lineage-negative mammary cell populations from 14-week-old *WT* and *PR*$^{-/-}$ littermates. Data= represent mean ±SD from 4 *PR*$^{+/+}$ and 4 *PR*$^{-/-}$ mice. Unpaired Student t-test, two-tailed. **b** Dot= plot showing percentage of Sma+ cells in contralateral transplants of *WT.EGFP*$^{+}$ and= *Wnt4*$^{-/-}$*.EGFP*$^{+}$ mammary epithelia derived for embryonic mammary buds, *P*=0.021. **c** Venn= diagram showing genes that are differentially expressed between *Wnt4* deleted (*MMTV::Cre*$^{+}$*.Wnt4*$^{fl/fl}$*. mT/mG*) and control (*MMTV::Cre*$^{+}$*.Wnt4*$^{wt/wt}$*.mT/mG*) lineage= depleted mammary cells, FACS sorted for GFP and separated into CD24 high/luminal and CD24 low/myoepithelial populations. **d**, **e** Volcano plot showing genes differentially expressed between *WT* and *Wnt4*$^{-/-}$ luminal (**d**) and myoepithelial (**e**) cells in 3= independent experiments; Kolmogorov–Smirnov test, genes above the dotted line have *P* values <0.05. Genes with fold change log2(FC)>0.5 are colored in red and log2(FC)<-0.5 in blue. Names of selected genes are indicated. **f**, **g** GSEA plots showing gene sets that are differentially regulated between *WT* and *Wnt4*$^{-/-}$ mammary epithelial cells, CD24 high or= CD24 low. **h** CNET plot of Reactome pathway enrichment analysis (ReactomePA) showing associations between cell functions and differentially-regulated genes in *WT* and *Wnt4*$^{-/-}$ CD24low cells; n=3, Kolmogorov–Smirnov test, genes with *P* values <0.05 are= shown. *, p<0.05; **, p<0.01; ***, p<0.001; ****, p<0.0001, n.s. not significant.

Supplementary Figure 2

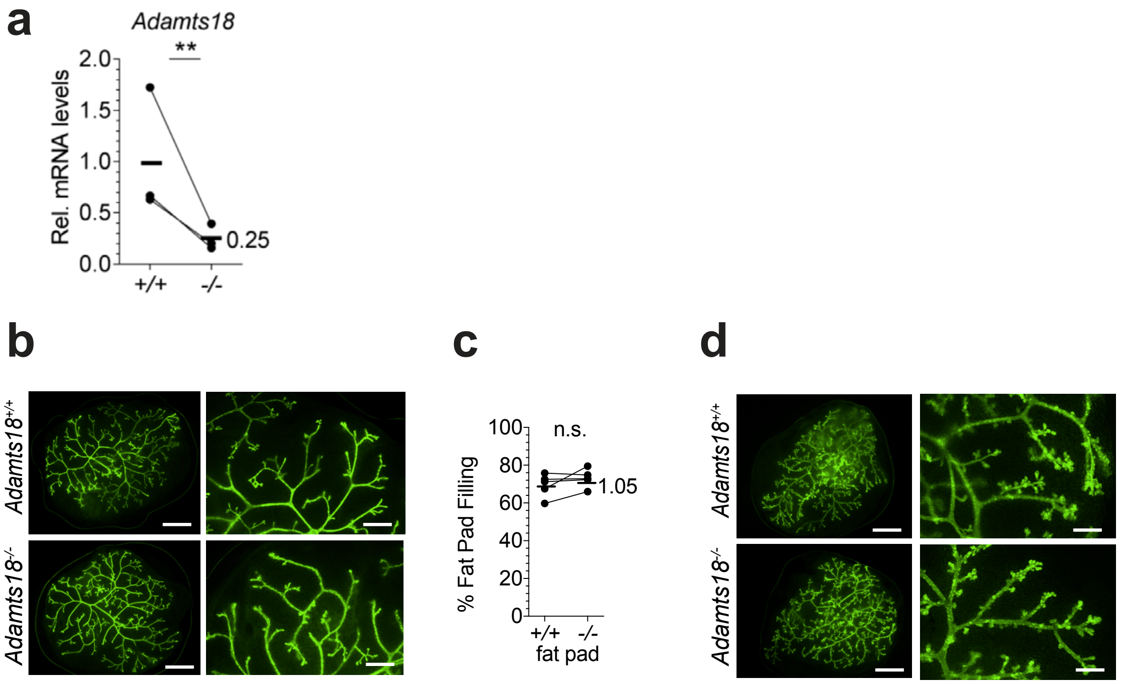

Supplementary Figure 2. *Adamts18* deletion in the mammary fat pad

a Dot plots showing relative transcript levels of *Adamts18* normalized to *Hprt* in 3 pairs of contra lateral glands transplanted with *WT* versus *Adamts18*$^{-/-}$ epithelium. Paired Student t-test, two-tailed. **b** Representative fluorescence stereo micrographs of 4 *WT* and 4 *Adamts18*$^{-/-}$ fat pads endowed with *WT.EGFP*+ epithelium and then transplanted unto the abdominal wall of 4 F1 recipient mice, 6 weeks after surgery. Scale bars, 5 mm, (left), 2 mm (right). **c** Dot plot showing percentage of filling of *WT* and *Adamts18*$^{-/-}$ fat pads with engrafted *WT.EGFP*+ epithelium. Paired Student t-test, two-tailed. **d** Representative fluorescence stereo micrographs of 6= *WT* and 6 *Adamts18*$^{-/-}$ fat pads with engrafted *WT.EGFP*+ epithelium and= transplanted unto abdomen of 6 F1 recipient mice 12 weeks after surgery. Scale= bars, 5 mm, left-hand panels, 2 mm right-hand panels. **, p<0.01; n.s. not significant.

Supplementary Figure 3

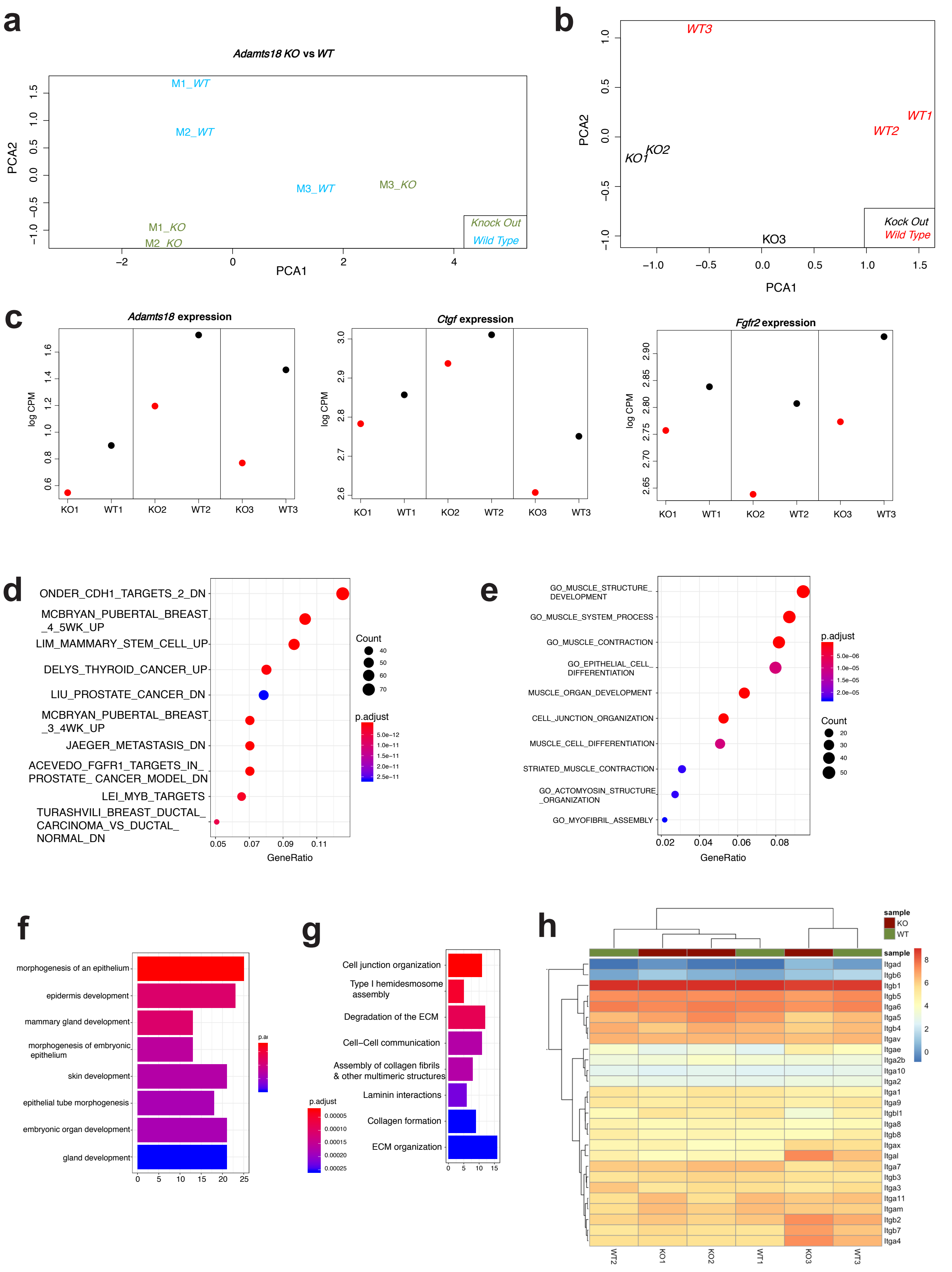

Supplementary Figure 3. Effects of *Adamts18* deletion on global gene expression

a PCA plot showing the two most significant principal components with samples labelled according to the *Adamts18* genotype of the engrafted epithelium. **b** Batch-corrected PCA plot showing the two most significant principal components with samples labelled according to the *Adamts18* genotype of the engrafted epithelium. **c** Dot plots showing counts for *Adamts18, Ctgf and Fgfr2* in contralateral glands transplanted with *WT* and *Adamts18*$^{-/-}$ epithelia (n=6). **d** Pathway enrichment analysis using ClusterProfiler for genes differentially expressed between contralateral glands transplanted with *WT* and *Adamts18*$^{-/-}$ epithelia (n=3). **e** GO enrichment analysis using ClusterProfiler for genes differentially expressed between contralateral glands transplanted with *WT* and *Adamts18*$^{-/-}$ epithelia (n=3). **f** GO enrichment analysis using ClusterProfiler on genes downregulated in contralaterally transplanted glands of *Adamts18*$^{-/-}$compared to *WT* epithelial cells (n=3). **g** Reactome pathway analysis (ReactomePA) of genes downregulated in contralateral glands transplanted with *Adamts18*$^{-/-}$ compared to *WT* epithelial cells (n=3). **h** Heatmap generated using log2 normalized counts of 27 *Itg* genes from RNA sequencing on contralateral glands transplanted with *WT* and *Adamts18*$^{-/-}$ epithelia; n=3.

Supplementary Figure 4

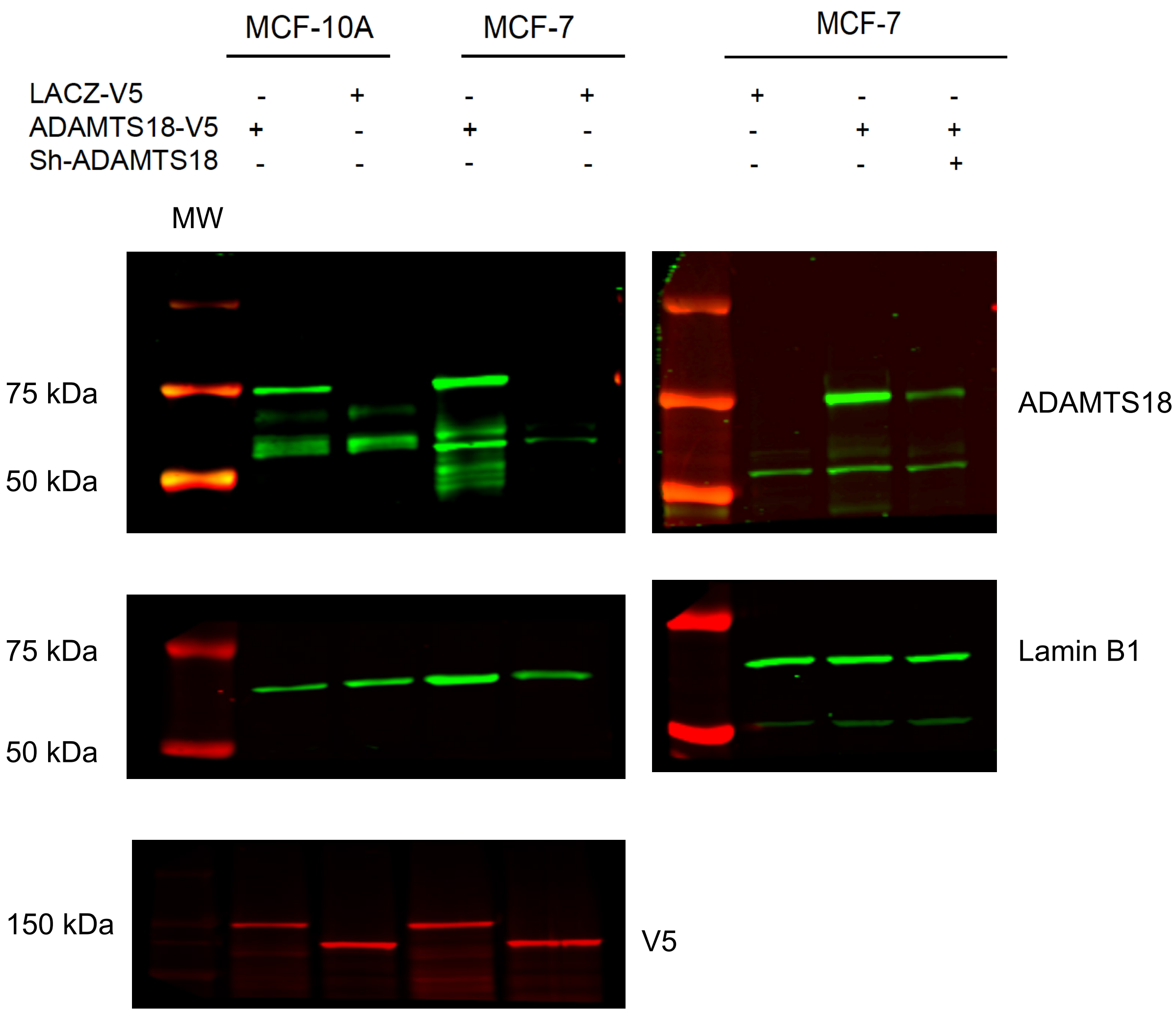

Supplementary Figure 4. Validation of anti-ADAMTS18 antibody
Western blots of ADAMTS18 protein from transfected MCF7 and MCF10A cells. Transfected expression vectors are indicated at the top left, antibodies on the right. LAMINB1 was used as a loading control. Three independent transfection experiments are shown.

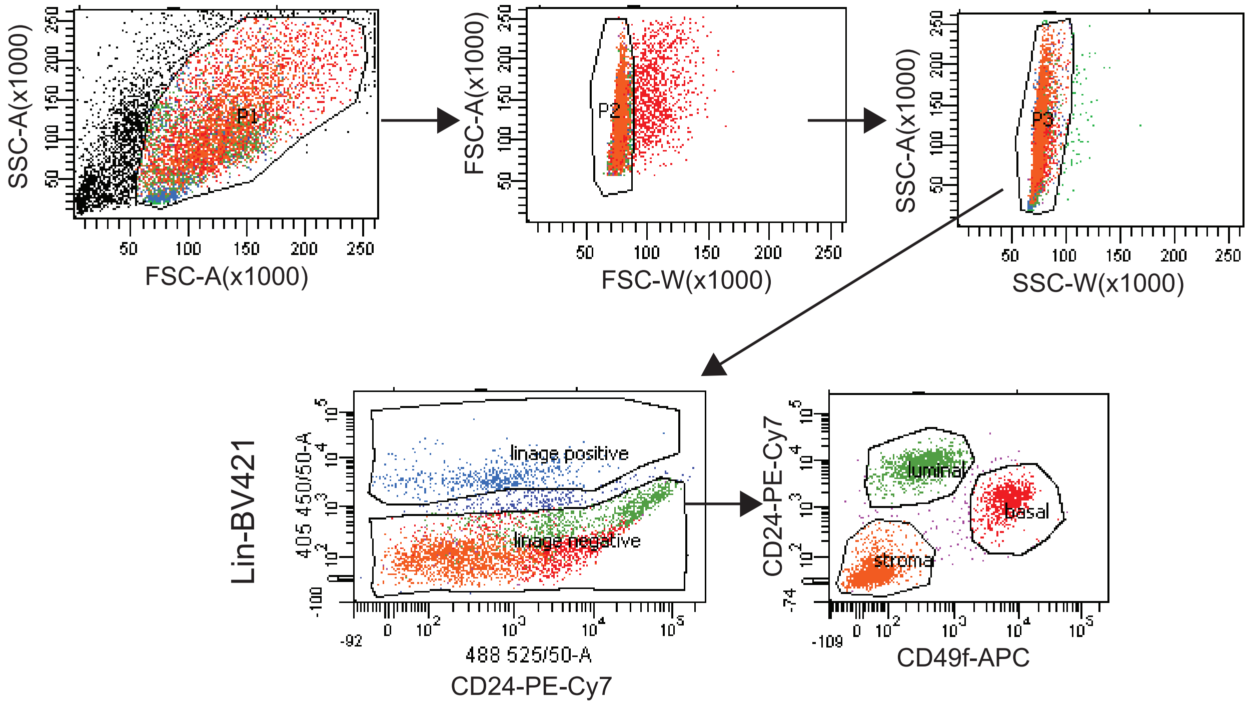

Supplementary Figure 5. Flow cytometry gating strategy

Scheme showing the strategy for the analysis of stromal, luminal, and myoepithelial cell populations from dissociated mammary glands.

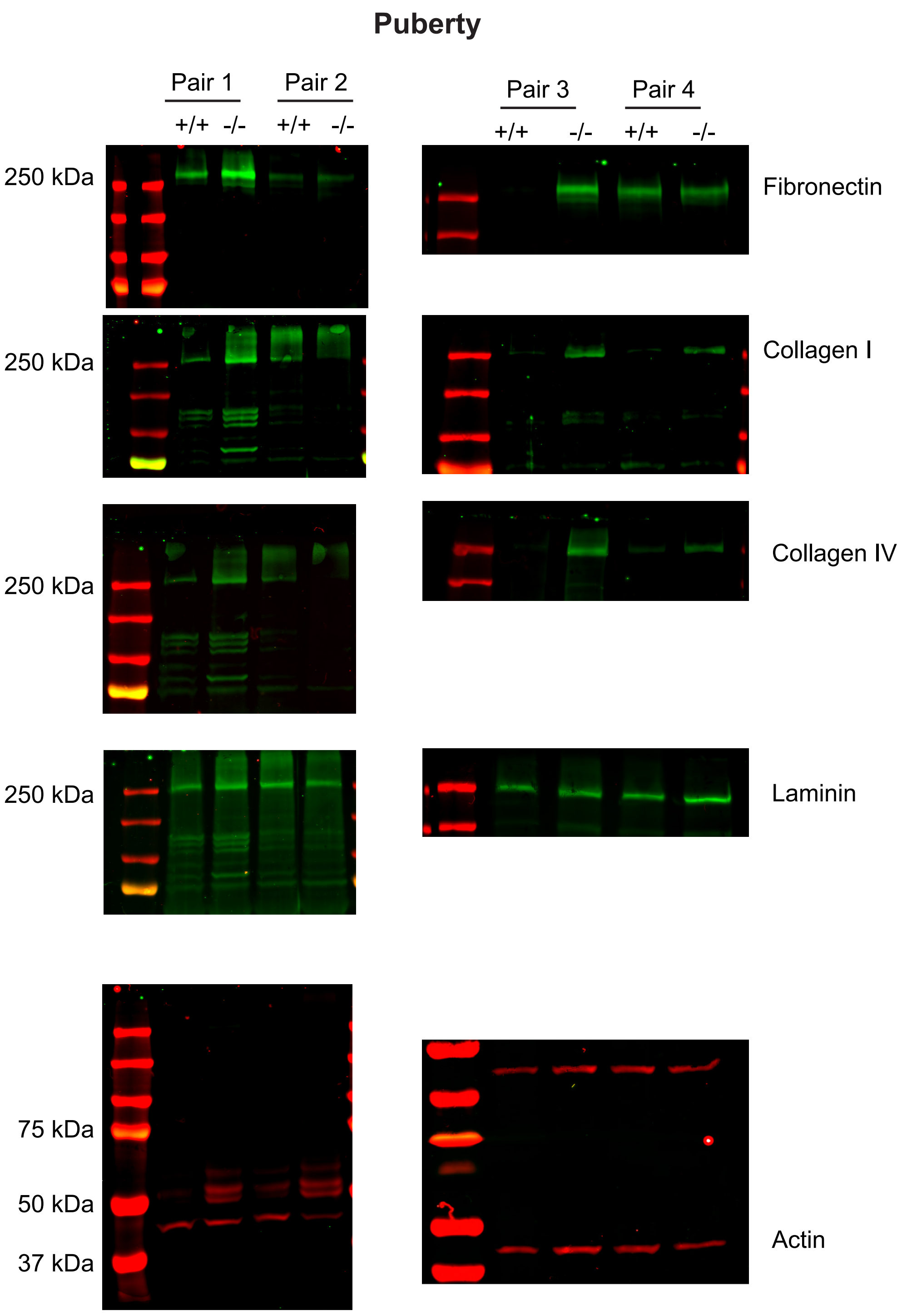

Supplementary Figure 6 related to Figure 6b. BM proteins and glycoproteins

Uncropped Western blot data of the different ECM proteins in pubertal *WT* and *Adamts18*$^{-/-}$ mice. Data represent 4 pairs of littermates.

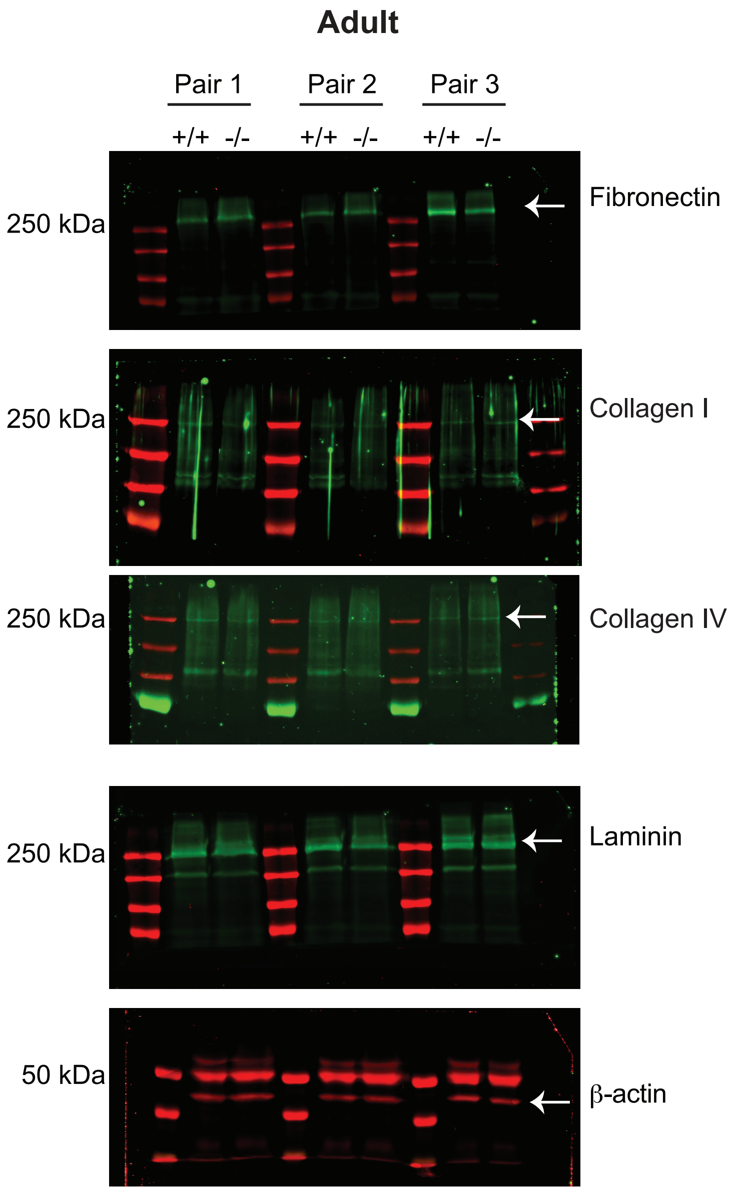

Supplementary Figure 7 related to Figure 6e. BM proteins and glycoproteins

Uncropped Western blot data of the different ECM proteins inadult *WT* and *Adamts18*$^{-/-}$ mice.

Data represent 3 pairs of littermates.

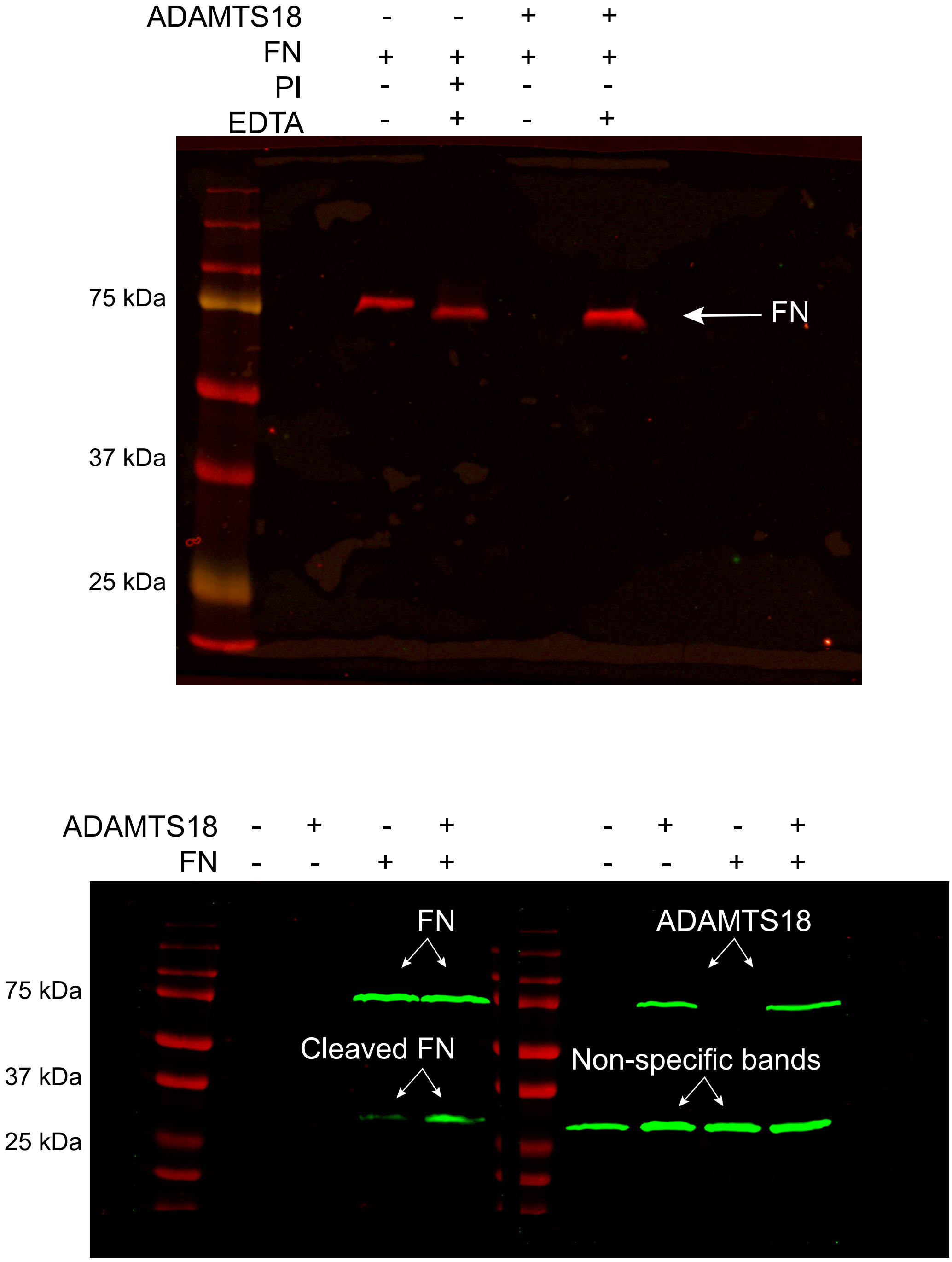

Supplementary Figure 8 related to Figure 6 i,h. BM proteins and glycoproteins

Uncropped Western blot data of Fibronectin cleavage by ADAMTS18.

Supplementary Table 1. Localization of ADAMTS18 interactors

Localization	Binding Proteins
Extracellular matrix	LAMB3, LAMA3 (Epiligrin), BETA-IG-H3, Cyr61, Vitronectin, Calnexin, LAMA4, PRSS11 (HtrA1), PGAR, LAMC2
Laminin-5 complex	LAMB3, LAMA3 (Epiligrin), LAMC2
Basement membrane	LAMB3, LAMA3 (Epiligrin), BETA-IG-H3, Vitronectin, LAMA4, LAMC2
Basal lamina	LAMB3, LAMA3 (Epiligrin), LAMA4, LAMC2
Extracellular matrix component	LAMB3, LAMA3 (Epiligrin), BETA-IG-H3, Vitronectin, LAMA4, LAMC2
Proteinaceous extracellular matrix	LAMB3, LAMA3 (Epiligrin), BETA-IG-H3, Cyr61, Vitronectin, LAMA4, PGAR, LAMC2
Laminin complex	LAMB3, LAMA3 (Epiligrin), LAMC2
Extracellular region part	LAMB3, AP complex 2 medium (mu) chain, DAF, LAMA3 (Epiligrin), ERp72, BETA-IG-H3, Cyr61, Vitronectin, PR65-alpha, Calnexin, LAMA4, SULF2, CRIM1, PRSS11 (HtrA1), PGAR, JWA, LAMC2, UGCGL1
Extracellular space	AP complex 2 medium (mu) chain, DAF, LAMA3 (Epiligrin), ERp72, BETA-IG-H3, Cyr61, Vitronectin, PR65-alpha, Calnexin, LAMA4, SULF2, CRIM1, PRSS11 (HtrA1), PGAR, JWA, LAMC2, UGCGL1
Extracellular region	LAMB3, AP complex 2 medium (mu) chain, DAF, LAMA3 (Epiligrin), ERp72, BETA-IG-H3, Cyr61, Vitronectin, PR65-alpha, Calnexin, LAMA4, Laminin 5, SULF2, CRIM1, PRSS11 (HtrA1), PGAR, JWA, LAMC2, UGCGL1
Endoplasmic reticulum lumen	ERp72, Cyr61, Vitronectin, Calnexin, UGCGL1
Extracellular exosome	AP complex 2 medium (mu) chain, DAF, LAMA3 (Epiligrin), BETA-IG-H3, Vitronectin, PR65-alpha, Calnexin, LAMA4, CRIM1, PRSS11 (HtrA1), JWA, UGCGL1
Extracellular vesicle	AP complex 2 medium (mu) chain, DAF, LAMA3 (Epiligrin), BETA-IG-H3, Vitronectin, PR65-alpha, Calnexin, LAMA4, CRIM1, PRSS11 (HtrA1), JWA, UGCGL1
Extracellular organelle	AP complex 2 medium (mu) chain, DAF, LAMA3 (Epiligrin), BETA-IG-H3, Vitronectin, PR65-alpha, Calnexin, LAMA4, CRIM1, PRSS11 (HtrA1), JWA, UGCGL1
Endomembrane system	AP complex 2 medium (mu) chain, DAF, ERp72, BETA-IG-H3, Cyr61, Vitronectin, RANBP7, Calnexin, UBR4, SULF2, NUP205, DHCR7, JWA, PRP12, UGCGL1
Smooth endoplasmic reticulum	ERp72, Calnexin
Vesicle	AP complex 2 medium (mu) chain, DAF, LAMA3 (Epiligrin), ERp72, BETA-IG-H3, Vitronectin, PR65-alpha, Calnexin, UBR4, LAMA4, CRIM1, PRSS11 (HtrA1), JWA, UGCGL1
Protein complex	LAMB3, AP complex 2 medium (mu) chain, LAMA3 (Epiligrin), ERp72, Vitronectin, RANBP7, Calnexin, NDUFA9, NUP205, DNAJA3 (TID1), LAMC2, UGCGL1
Intracellular organelle part	AP complex 2 medium (mu) chain, DAF, ERp72, BETA-IG-H3, Cyr61, Vitronectin, PR65-alpha, RANBP7, Calnexin, UBR4, NDUFA9, SULF2, NUP205, DDX27, DNAJA3 (TID1), DHCR7, RPS6, JWA, DLG5(P-dlg), PRP12, RBM34, UGCGL1
Laminin-2 complex	LAMC2

MetaCore-ADAMTS18 binding proteins localization

Supplementary Table 2. Metacore processes related to ADAMTS18 interactors

Processes	Binding Proteins
Extracellular matrix organization	LAMB3, LAMA3 (Epiligrin), BETA-IG-H3, Cyr61, Vitronectin, LAMA4, Laminin 5, SULF2, PRSS11 (HtrA1), LAMC2
Extracellular structure organization	LAMB3, LAMA3 (Epiligrin), BETA-IG-H3, Cyr61, Vitronectin, LAMA4, Laminin 5, SULF2, PRSS11 (HtrA1), LAMC2
Hemidesmosome assembly	LAMB3, LAMA3 (Epiligrin), Laminin 5, LAMC2
Extracellular matrix disassembly	LAMB3, LAMA3 (Epiligrin), Laminin 5, PRSS11 (HtrA1), LAMC2
Cellular component disassembly	LAMB3, LAMA3 (Epiligrin), PP2A structural, PR65-alpha, Laminin 5, NUP205, PRSS11 (HtrA1), LAMC2
Regulation of cell adhesion	DAF, LAMA3 (Epiligrin), PP2A structural, BETA-IG-H3, Cyr61, Vitronectin, PR65-alpha, LAMA4, Laminin 5, DNAJA3 (TID1)
Meiotic spindle elongation	PP2A structural, PR65-alpha
Cell-substrate junction assembly	LAMB3, LAMA3 (Epiligrin), Laminin 5, LAMC2
Endodermal cell differentiation	LAMB3, LAMA3 (Epiligrin), Vitronectin, Laminin 5
Protein folding	BAG-2, ERp72, Calnexin, DNAJA3 (TID1), N-glycanase 1, UGCGL1
Endoderm formation	LAMB3, LAMA3 (Epiligrin), Vitronectin, Laminin 5
Cell junction assembly	LAMB3, LAMA3 (Epiligrin), Laminin 5, DLG5(P-dlg), LAMC2
Cell adhesion	LAMB3, LAMA3 (Epiligrin), BETA-IG-H3, Cyr61, Vitronectin, LAMA4, Laminin 5, DLG5(P-dlg), LAMC2
Biological adhesion	LAMB3, LAMA3 (Epiligrin), BETA-IG-H3, Cyr61, Vitronectin, LAMA4, Laminin 5, DLG5(P-dlg), LAMC2
Cell junction organization	LAMB3, LAMA3 (Epiligrin), Laminin 5, DLG5(P-dlg), LAMC2
Gastrulation	LAMB3, LAMA3 (Epiligrin), Vitronectin, Laminin 5, RPS6
Mitotic sister chromatid separation	PP2A structural, PR65-alpha
Brown fat cell differentiation	LAMB3, LAMA4, Laminin 5
Endoderm development	LAMB3, LAMA3 (Epiligrin), Vitronectin, Laminin 5
Basement membrane assembly	Laminin 5, LAMC2
Apoptotic process involved in morphogenesis	PP2A structural, Cyr61

Supplementary Table 3. Genes with decreased expression in mammary glands engrafted with *Adamts18*$^{-/-}$ vesus *WT* epithelia; Reactome analysis.

Reactome Analysis Results

Pathway name	Entities found	Entities Total	Entities p Value	Entities FDR
Attenuation phase	14	47	1.05E-10	6.73E-08
HSF1-dependent transactivation	14	59	1.89E-09	6.03E-07
HSF1 activation	12	43	4.67E-09	9.90E-07
Cell junction organization	15	94	8.96E-08	1.42E-05
Type I hemidesmosome assembly	6	11	8.02E-07	1.02E-04
Regulation of HSF1-mediated heat shock response	14	113	4.46E-06	4.73E-04
Cell-Cell communication	15	133	6.24E-06	5.67E-04
Assembly of collagen fibrils and other multimeric structures	10	67	2.22E-05	1.75E-03
Laminin interactions	7	31	3.00E-05	1.98E-03
Cellular response to heat stress	14	135	3.14E-05	1.98E-03
Signaling by FGFR2 IIIa TM	6	24	6.41E-05	3.70E-03
Anchoring fibril formation	5	15	7.00E-05	3.70E-03
RUNX3 regulates YAP1-mediated transcription	4	9	1.28E-04	6.25E-03
Collagen formation	11	104	1.84E-04	8.28E-03
Collagen chain trimerization	7	44	2.57E-04	1.08E-02
Degradation of the extracellular matrix	13	148	2.98E-04	1.16E-02
FGFR2 mutant receptor activation	7	49	4.86E-04	1.80E-02
Formation of the cornified envelope	12	138	5.49E-04	1.92E-02
Cell-cell junction organization	8	67	6.33E-04	2.09E-02
Phospholipase C-mediated cascade; FGFR2	5	25	7.18E-04	2.22E-02
Collagen degradation	8	69	7.64E-04	2.29E-02
FGFR2 ligand binding and activation	5	26	8.54E-04	2.48E-02
YAP1- and WWTR1 (TAZ)-stimulated gene expression	4	18	1.68E-03	4.54E-02
PI-3K cascade:FGFR2	5	31	1.84E-03	4.79E-02
Signaling by FGFR2 in disease	7	63	2.04E-03	4.85E-02
SHC-mediated cascade:FGFR2	5	32	2.11E-03	4.85E-02
MET activates PTK2 signaling	5	32	2.11E-03	4.85E-02
Extracellular matrix organization	19	329	2.31E-03	5.08E-02
FRS-mediated FGFR2 signaling	5	33	2.41E-03	5.30E-02
Downregulation of ERBB4 signaling	3	10	2.82E-03	5.93E-02
Apoptotic cleavage of cell adhesion proteins	3	11	3.68E-03	7.36E-02
Smooth Muscle Contraction	6	55	4.58E-03	8.43E-02
Sema4D induced cell migration and growth-cone collapse	4	24	4.67E-03	8.43E-02
Activation of the TFAP2 (AP-2) family of transcription factors	3	12	4.68E-03	8.43E-02
Collagen biosynthesis and modifying enzymes	7	76	5.62E-03	9.25E-02
Antagonism of Activin by Follistatin	2	4	5.63E-03	9.25E-02
Tandem of pore domain in a weak inwardly rectifying K+ channels	2	4	5.63E-03	9.25E-02
Downstream signaling of activated FGFR2	5	41	5.97E-03	9.25E-02
Negative regulation of FGFR2 signaling	5	41	5.97E-03	9.25E-02
NOTCH4 Intracellular Domain Regulates Transcription	4	26	6.17E-03	9.25E-02
ECM proteoglycans	7	79	6.88E-03	1.00E-01

Supplementary Table 4. List of primary and secondary antibodies used in this study.

Antibodies	Source	Identifier	Clone
pHH3	Millipore	06-570	3H10
Cleaved caspase 3	Cell Signaling	N66645	Asp175
Yap	Cell Signaling	4912S	N/A
Sma	Neo markers	MS-113-P	1A4
Sma	Thermo Scientific	Rb-9010-P1	N/A
ERα	Santa Cruz	MC20	N/A
PR	Thermo Scientific	MA5-14505	SP2
CK7	Abcam	ab183344	SP52
P63	Bio genex	MU418-UC	4A4
LaminB1	Abcam	AB16048	N/A
Fibronectin	Santa Cruz Biotechnology	SC-81767	616
Fibronectin	Abcam	AB2413	N/A
Laminin	Abcam	AB30320	N/A
Collagen I	Abcam	AB34710	N/A
Collagen IV	Abcam	AB6586	N/A
β-actin	Sigma Aldrich	mab1501	C4
anti-CD24-PE-Cy7	BD Pharmingen	560536	N/A
anti-CD49f-APC	Biolegend	313616	GoH3
anti-CD31-BV421	BD Pharmingen	563356	390 (RUO)
anti-CD45-BV421	BD Pharmingen	563890	30 F11 (RUO)
anti-Ter119-BV421	BD Pharmingen	563998	TER-119 (RUO)
Anti-V5	Sigma	A7345	v5-10
Mouse Alexa 488	ThermoFisher Scientific	A-11029	N/A
Mouse Alexa 488	ThermoFisher Scientific	A-10037	N/A
Rabbit Alexa 488	ThermoFisher Scientific	A-21206	N/A
Rabbit Alexa 568	ThermoFisher Scientific	A-10042	N/A
Mouse HRP	Jackson ImmunoResearch	715-035-150	N/A
Rabbit HRP	Jackson ImmunoResearch	711-035-152	N/A

Supplementary Table 5. List of primer sequences with their references

Primers	Forward and Reverse Sequences	References
Adamts18 mouse specific	5'-GGACAGATTTATGATGCCGACA-3', 5'-GGTGGCACCAGAGTGACTT-3'	1-3
Gata3	5'-CTCGGCCATTCGTACATGGAA-3', 5'-GGATACCTCTGCACCGTAGC-3'	1-3
Adamts16	5'-CCCCCATTCTCTGGGTCCA-3', 5'-CTGGGTCACAGCTCTTCTCC-3'	1-3
Wnt4	5'-AGGAGTGCCAATACCAGTTCC-3', 5'-TGTGAGAAGGCTACGCCATA-3'	4
Wnt5A	5’-CAATGAAGCAGGCCGTAGGACAGT-3’ 5’-CATGGCCGCCGCGCTATCAT-3’	N/A
Wnt7B	5’-GGGAGAAGCAAGGCTACTAC-3’ 5’-CCAGCAAGTTTTGGTGGTAT-3’	N/A
Wnt10A	5’-CATGAGTCCCAGCATCAGTT-3’, 5’-GCCTTCAGTTTACCCAGAGC-3’	N/A
Wnt11	5’-GGATCCCAAGCCAATAAACT-3’ 5’-GGTAGCGGGTCTTGAGGTCA-3’	N/A
LGR5	5’-CCTACTCGAAGACTTACCCAG-3’ 5’-GCATTCGGGTGAATGATAGCA-3’	N/A
LRP5	5’-GTGTGCAGTTGCAGGACAAT-3’ 5’-CATCATCGGTCCAGTACACG-3’	N/A
LRP6	5’-GGGCAGACACAGGAACAAAT-3’ 5’-GCGTTCACTTCCATCCATAG-3’	N/A
ITGA3	5'-CACGCACATCATCACTGTTG-3' 5'-CTGCCACCCATCATTGTTCA-3'	5
ITGB4	5’-CTCCTCTCCTAGCGCCTTCT-3’ 5’-CACCTACCCCTGCTCTCTGA-3’	5
ITGB7	5’- CAGCTCATCATGGATGCTTA-3’ 5’-GAAGAAGAACAGCTGGTTGTC-3’	6
Axin2	5'-GGCAGTGATGGAGGAAAATG-3', 5'-TGGGTGAGAGTTTGCACTTG-3'	4
Ck18	5'-CAAGATCATCGAAGACCTGAGGGC-3', 5'-TGTTCATACTGGGCACGGATGTCC-3'	7
Adamts18	5'-TGTCGTGCCAGTAGAAGTGG-3', 5'-AGCACCGTCCTTTCCAAGTA-3'	8
Ctgf	5'-TGTGCACTGCCAAAGATGGTGCAC-3', 5'-TGGGCAGGCGCACGTCCATG-3'	9

Fgfr2	5'-CCTGCGGAGACAGGTAACAG-3',<br>5'-CGCGTTGTTATCCTCACCA-3'	10
Ck5	5'-GACCAGTCAACATCTCTGTC-3',<br>5'-TGCCAACACCAATGCTGCTG-3'	N/A
Hprt	5'-CAAACTTTGCTTTCCCTGGT-3',<br>5'-CAAGGGCATATCCAACAACA-3'	N/A
Wnt1	5'-TGCTTTTAGTGCCGCCCGGG-3',<br>5'-CACGATGCCCCACCATCGGC-3'	N/A
Wap	5'-GTTGCCTCATCAGCCTTGTT-3',<br>5'-CGTTGGTTTGGCAGATGATA-3'	N/A
Lalba	5'-GACAACGGCAGCACAGAGTA-3',<br>5'-TCTTCTTGGCACACGCTATG-3'	N/A
Csna	5'-GCACCTCTGTGGAGGAAATC-3',<br>5'-GTGAGCATTTGGGAAGCTCT-3'	N/A
Fn1	5'-AATGGAAAAGGGGAATGGAC-3',<br>5'-CTCGGTTGTCCTTCTTGCTC-3'	N/A
GAPDH	5'-AGGGCTGCTTTTAACTCTGGT-3',<br>5'-CCCCACTTGATTTTGGAGGGA-3'	11
ADAMTS18	5'-ACACTGCAATAACCCCAAGC-3',<br>5'-TAGAACCATCCACGGAAAGG-3'	N/A

Supplementary Methods. Bioinformatic analysis

RNA-seq. RNA was extracted from GFP+ FACS-sorted cells using miRNeasy Mini Kit (Qiagen). Libraries were prepared in 2 steps and sequenced on Illumina NextSeq 500 instrument with single-end reads of 85nt. Base calls and Illumina adaptors trimming performed using bcl2fastq v2.18. Clontech adaptors trimming performed with CLC 9.Raw reads were aligned to the mouse genome (mm9) using TopHat (v2.0.11)[12], the exact parameters were: tophat -p 6 -g 2 --no-novel-juncs --no-novel-indels --b2-sensitive. Gene counts were generated using FeatureCounts[13]. Data preprocessing was done using the edgeR package from Bioconductor[14]. The Voom function[15] of the limma package from Bioconductor[16] was used to normalize the data for sequencing depth differences, estimate the mean-variance relationship of the log-counts, and generate a precision weight for each observation so that data were ready for the limma linear fitting function (lmFit). Batch effects corresponding to different mice were modeled via the design matrix specifications. Genes were considered differentially expressed based on p-value cutoff ($p<0.05$) and fold-change ($|\log_2(FC)| > 0.32$). Pathway enrichment analysis was carried out using ReactomePA and ClusterProfiler from Bioconductor used with default parameters. For GO enrichment analysis the genome wide annotation for human (org.Hs.eg.db) Bionconductor package as well as the C2 and C5 curated gene set collections from the MSigDB v6.2[17,18] Broad Institute were used. Reactome on-line resource (http://www.reactome.org) was used to generate the results shown in Supplementary Table 5.

Affymetrix microarrays mouse Gene 1.0 ST. Affimetrix gene expression analysis was carried out using the mouse Gene 1.0 ST chip. Four cell extracts were used for the microarray analysis: FACS-sorted GFP+ luminal and myoepithelial cells from conditionally *Wnt4* deleted (*MMTV::Cre*$^{+}$.*Wnt4*$^{fl/fl}$.*mT/mG*) and control (*MMTV::Cre*$^{+}$.*Wnt4*$^{wt/wt}$.*mT/mG*) epithelia. The analysis of Affymetrix arrays starts with CEL files. These are the result of the processing of the raw image files using the Affymetrix software and contain estimated probe intensity values. Data were calibrated using the full RMA algorithm (Robust MultiArray Average)[19] from Bioconductor in order to background-correct, normalize and summarize. Subsequently, the limma package from Bioconductor was used for differential expression analysis.

Supplementary References

1. Spandidos, A. *et al.* A comprehensive collection of experimentally validated primers for Polymerase Chain Reaction quantitation of murine transcript abundance. *BMC Genomics* **9**, 633 (2008).
2. Spandidos, A., Wang, X., Wang, H. & Seed, B. PrimerBank: a resource of human and mouse PCR primer pairs for gene expression detection and quantification. *Nucleic Acids Research* **38**, D792–D799 (2010).
3. Wang, X. A PCR primer bank for quantitative gene expression analysis. *Nucleic Acids Research* **31**, 154e–1154 (2003).
4. Rajaram, R. D. *et al.* Progesterone and Wnt4 control mammary stem cells via myoepithelial crosstalk. *The EMBO Journal* **34**, 641–652 (2015).
5. Romagnoli, M. *et al.* Deciphering the Mammary Stem Cell Niche: A Role for Laminin-Binding Integrins. *Stem Cell Reports* **12**, 831–844 (2019).
6. Desiderio, U. V., Zhu, X. & Evans, J. P. ADAM2 interactions with mouse eggs and cell lines expressing α4/α9 (ITGA4/ITGA9) integrins: implications for integrin-based adhesion and fertilization. *PLoS ONE* **5**, e13744 (2010).
7. Ayyanan, A. *et al.* Perinatal Exposure to Bisphenol A Increases Adult Mammary Gland Progesterone Response and Cell Number. *Molecular Endocrinology* **25**, 1915–1923 (2011).
8. Ataca, D. *et al. Adamts18* deletion results in distinct developmental defects and provides a model for congenital disorders of lens, lung, and female reproductive tract development. *Biology Open* **5**, 1585–1594 (2016).
9. Cotton, J. L. *et al.* YAP/TAZ and Hedgehog Coordinate Growth and Patterning in Gastrointestinal Mesenchyme. *Developmental Cell* **43**, 35-47.e4 (2017).
10. Pond, A. C. *et al.* Fibroblast Growth Factor Receptor Signaling Is Essential for Normal Mammary Gland Development and Stem Cell Function. *STEM CELLS* **31**, 178–189 (2013).
11. Tanooka, H. *et al.* Mutant mouse p53 transgene elevates the chemical induction of tumors that respond to gene silencing with siRNA. *Cancer Gene Therapy* **17**, 1–10 (2010).
12. Kim, D. et al. TopHat2: accurate alignment of transcriptomes in the presence of insertions, deletions and gene fusions. *Genome Biology* **14**, R36 (2013).
13. Liao, Y., Smyth, G. K. & Shi, W. featureCounts: an efficient general purpose program for assigning sequence reads to genomic features. *Bioinformatics* **30**, 923–930 (2014).
14. Robinson, M. D., McCarthy, D. J. & Smyth, G. K. edgeR: a Bioconductor package for differential expression analysis of digital gene expression data. *Bioinformatics* **26**, 139–140 (2010).
15. Law, C. W., Chen, Y., Shi, W. & Smyth, G. K. voom: precision weights unlock linear model analysis tools for RNA-seq read counts. *Genome Biology* **15**, R29 (2014).
16. Ritchie, M. E. et al. limma powers differential expression analyses for RNA-sequencing and microarray studies. *Nucleic Acids Research* **43**, e47–e47 (2015).
17. Liberzon, A. et al. Molecular signatures database (MSigDB) 3.0. *Bioinformatics* **27**, 1739–1740 (2011).
18. Subramanian, A. et al. Gene set enrichment analysis: A knowledge-based approach for interpreting genome-wide expression profiles. *Proceedings of the National Academy of Sciences* **102**, 15545–15550 (2005).
19. Irizarry, R. A. et al. Exploration, normalization, and summaries of high density oligonucleotide array probe level data. *Biostatistics* **4**, 249–264 (2003).
