# Supplementary material for: The secreted protease Adamts18 links hormone action to activation of the mammary stem cell niche
Source: Nat Commun. 2020 Mar 26;11:1571. doi: 10.1038/s41467-020-15357-y (PMC7099066; doi:10.1038/s41467-020-15357-y)
Supplement: Supplementary file 3 — Description of Additional Supplementary Files [file 41467_2020_15357_MOESM3_ESM.docx]

Description of Additional Supplementary Files

File name: Supplementary Data 1

Description: ADAMTS18 interactors
