# Supplementary material for: The secreted protease Adamts18 links hormone action to activation of the mammary stem cell niche
Source: Nat Commun. 2020 Mar 26;11:1571. doi: 10.1038/s41467-020-15357-y (PMC7099066; doi:10.1038/s41467-020-15357-y)
Supplement: Supplementary file 5 — Reporting Summary [file 41467_2020_15357_MOESM5_ESM.pdf]

nature research

Corresponding author(s): Cathrin Brisken, MD PhD

Last updated by author(s): Feb 14, 2020

Reporting Summary

Nature Research wishes to improve the reproducibility of the work that we publish. This form provides structure for consistency and transparency in reporting. For further information on Nature Research policies, see Authors & Referees and the Editorial Policy Checklist .

Statistics

For all statistical analyses, confirm that the following items are present in the figure legend, table legend, main text, or Methods section.

n/a	Confirmed	
☐	☒	The exact sample size (*n*) for each experimental group/condition, given as a discrete number and unit of measurement
☐	☒	A statement on whether measurements were taken from distinct samples or whether the same sample was measured repeatedly
☐	☒	The statistical test(s) used AND whether they are one- or two-sided<br>*Only common tests should be described solely by name; describe more complex techniques in the Methods section.*
☒	☐	A description of all covariates tested
☒	☐	A description of any assumptions or corrections, such as tests of normality and adjustment for multiple comparisons
☐	☒	A full description of the statistical parameters including central tendency (e.g. means) or other basic estimates (e.g. regression coefficient) AND variation (e.g. standard deviation) or associated estimates of uncertainty (e.g. confidence intervals)
☐	☒	For null hypothesis testing, the test statistic (e.g. *F*, *t*, *r*) with confidence intervals, effect sizes, degrees of freedom and *P* value noted<br>*Give P values as exact values whenever suitable.*
☒	☐	For Bayesian analysis, information on the choice of priors and Markov chain Monte Carlo settings
☒	☐	For hierarchical and complex designs, identification of the appropriate level for tests and full reporting of outcomes
☒	☐	Estimates of effect sizes (e.g. Cohen's *d*, Pearson's *r*), indicating how they were calculated

Our web collection on statistics for biologists contains articles on many of the points above.

Software and code

Policy information about availability of computer code

Data collection	No software or code were used for data collection
Data analysis	PRISM (version 6) https://www.graphpad.com/<br>FlowJo (version 9.8.2) https://www.flowjo.com<br>Fiji/ImageJ (version 1.52t) https://imagej.nih.gov/ij/<br>QuPath (v0.1.4) https://qupath.github.io/<br>MetaCoreTM https://portal.genego.com/<br>TopHat (v2.0.11) https://ccb.jhu.edu/software/tophat<br>Affy package (version 1.60.0) https://www.bioconductor.org<br>Feature Counts (version 1.6.2) https://www.bioconductor.org<br>Oligo (1.46.0) https://www.bioconductor.org<br>Biobase (v2.42.0) https://www.bioconductor.org<br>EdgeR (v3.24.0) https://www.bioconductor.org<br>Limma (v3.38.0) https://www.bioconductor.org<br>ClusterProfiler (v3.14.3) https://www.bioconductor.org<br>ReactomePA (1.25.1) https://www.bioconductor.org<br>Reactome (version 70) http://www.reactome.org<br>Scaffold Proteome Software (http://www.proteomesoftware.com/)

For manuscripts utilizing custom algorithms or software that are central to the research but not yet described in published literature, software must be made available to editors/reviewers. We strongly encourage code deposition in a community repository (e.g. GitHub). See the Nature Research guidelines for submitting code & software for further information.

Data

Policy information about availability of data

All manuscripts must include a data availability statement. This statement should provide the following information, where applicable:

- Accession codes, unique identifiers, or web links for publicly available datasets
- A list of figures that have associated raw data
- A description of any restrictions on data availability

The datasets generated during and/or analysed during the current study are available from the corresponding author on reasonable request. The datasets generated and analysed during the current study are available in GEO under the accession numbers GSE145717 for microarray data and GSE145680 for RNA sequencing. Western blot raw data can be found in the supplementary figures 4-7.

Field-specific reporting

Please select the one below that is the best fit for your research. If you are not sure, read the appropriate sections before making your selection.

☒ Life sciences ☐ Behavioural & social sciences ☐ Ecological, evolutionary & environmental sciences

For a reference copy of the document with all sections, see nature.com/documents/nr-reporting-summary-flat.pdf

Life sciences study design

All studies must disclose on these points even when the disclosure is negative.

Sample size	No statistical test was used to estimate the sample size. Sample size was estimated based on previous experience with the experimental approaches (Rajaram, R. et al. 2015 and Cagnet, S. et al. 2018).
Data exclusions	No data were excluded
Replication	All experiments were performed at least 3 independent times, with the exception of the proteomics analysis which was performed twice. All attempts replication were successful.
Randomization	This study does not include comparison studies among groups, so this point is not applicable.
Blinding	Image analysis in Figure 2k, 2m, 3i, and 3j was carried out by "blinded" experimentator i.e. the experimentator did not know the corresponding genotype. For the rest of the experiments, investigators were not blinded to group allocation.

Reporting for specific materials, systems and methods

We require information from authors about some types of materials, experimental systems and methods used in many studies. Here, indicate whether each material, system or method listed is relevant to your study. If you are not sure if a list item applies to your research, read the appropriate section before selecting a response.

Materials & experimental systems

n/a	Involved in the study	
☐	☒	Antibodies
☐	☒	Eukaryotic cell lines
☒	☐	Palaeontology
☐	☒	Animals and other organisms
☐	☒	Human research participants
☒	☐	Clinical data

Methods

n/a	Involved in the study	
☒	☐	ChIP-seq
☐	☒	Flow cytometry
☒	☐	MRI-based neuroimaging

Antibodies

Antibodies used	Primary antibodies used in this study: pHH3 (ser10) (Millipore, 06-570, 3H10, https://www.merckmillipore.com/CH/de/product/Anti-phospho-Histone-H3-Ser10-Antibody-Mitosis-Marker,MM_NF-06-570?ReferrerURL=https%3A%2F%2Fwww.google.com%2F); Cleaved caspase-3 (Cell Signaling, 9661S, Asp175, https://www.cellsignal.com/products/primary-antibodies/cleaved-caspase-3-asp175-antibody/9661);Yap (Cell signaling, 4912S, https://www.cellsignal.com/products/primary-antibodies/yap-antibody/4912); SMA (Thermo Scientific, MS-113-P, 1A4, https://assets.fishersci.com/TFS-Assets/APD/Specification-Sheets/D11616~.pdf); SMA (Thermo Scientific, RB-9010-P1 https://assets.fishersci.com/TFS-Assets/APD/Specification-Sheets/D12461~.pdf); ER (Santa Cruz, sc-542, MC20, https://www.scbt.com/p/eralpha-antibody-mc-20); PR (Thermo Scientific, MA5-14505, SP2, https://www.thermofisher.com/antibody/product/Progesterone-Receptor-Antibody-clone-SP2-Monoclonal/MA5-14505); CK7 (Abcam, ab183344, SP52, https://www.abcam.com/cytokeratin-7-antibody-sp52-ab183344.html); p63 (BioGenex, MU418-UC, 4A4, http://store.biogenex.com/international/anti-p63.html); Lamin B1 (Abcam, ab16048, https://

www.abcam.com/lamin-b1-antibody-nuclear-envelope-marker-ab16048.html); Fibronectin (Santa Cruz, sc-81767, 616, https://www.scbt.com/p/fibronectin-antibody-616); Fibronectin (Abcam, ab2413, https://www.abcam.com/fibronectin-antibody-ab2413.html); Laminin (Abcam, ab30320); CollagenI (Abcam, ab34710, https://www.abcam.com/collagen-i-antibody-ab34710.html); CollagenIV (Abcam, ab6586, https://www.abcam.com/collagen-iv-antibody-ab6586.html); Actin (Sigma Aldrich, MAB1501, C4, https://www.sigmaaldrich.com/catalog/product/mm/mab1501?lang=fr&region=CH); Anti-CD24-PE-Cy7 (BD Pharmingen,560536, https://www.bdbiosciences.com/us/applications/research/stem-cell-research/cancer-research/mouse/pe-cy7-rat-anti-mouse-cd24-m169/p/560536); Anti-CD49f-APC (Biolegend, 313616, GoH3, https://www.biolegend.com/en-us/products/apc-anti-human-mouse-cd49f-antibody-5617); Anti-CD31-BV421 (BD Pharmingen, 563356, 390 (RUO), https://www.bdbiosciences.com/us/applications/research/stem-cell-research/cancer-research/mouse/bv421-rat-anti-mouse-cd31-390/p/563356); Anti-CD45-BV421 (BD Pharmingen, 563890, 30 F11 (RUO), https://www.bdbiosciences.com/us/reagents/research/antibodies-buffers/immunology-reagents/anti-mouse-antibodies/cell-surface-antigens/bv421-rat-anti-mouse-cd45-30-f11/p/563890); Anti-Ter119-BV421 (BD Pharmingen, 563998, TER-119 (RUO), https://www.bdbiosciences.com/eu/applications/research/stem-cell-research/mesoderm-markers/mouse/bv421-rat-anti-mouse-ter-119erythroid-cells-ter-119/p/563998); Anti-V5 (Sigma, A7345, v5-10, https://www.sigmaaldrich.com/catalog/product/sigma/a7345?lang=fr&region=CH).

Secondary Antibodies used in this study: Mouse Alexa 488 (ThermoFisher Scientific, A-11029, https://www.thermofisher.com/antibody/product/Goat-anti-Mouse-IgG-H-L-Highly-Cross-Adsorbed-Secondary-Antibody-Polyclonal/A-11029); Mouse Alexa 568 (ThermoFisher Scientific,A-10037, https://www.thermofisher.com/antibody/product/Donkey-anti-Mouse-IgG-H-L-Highly-Cross-Adsorbed-Secondary-Antibody-Polyclonal/A10037); Rabbit Alexa 488 (ThermoFisher Scientific, A-21206, https://www.thermofisher.com/antibody/product/Donkey-anti-Rabbit-IgG-H-L-Highly-Cross-Adsorbed-Secondary-Antibody-Polyclonal/A-21206); Rabbit Alexa 568 (ThermoFisher Scientific, A-10042, https://www.thermofisher.com/antibody/product/Donkey-anti-Rabbit-IgG-H-L-Highly-Cross-Adsorbed-Secondary-Antibody-Polyclonal/A10042); Mouse HRP (Jackson ImmunoResearch, 715-035-150, https://www.jacksonimmuno.com/catalog/products/715-035-150); Rabbit HRP, Jackson Immunosearch, 711-035-152, https://www.jacksonimmuno.com/catalog/products/711-035-152).

Validation

The following antibodies: SMA (rabbit and mouse), ER, PR, p63, anti-CD24-PE-Cy7, anti-CD49f-APC, anti-CD31-BV421, and anti-Ter119-BV421 have been previously validated in (Cagnet, S. et al. 2018). Lamin B1 and Actin were previously validated in (Sflomos, G. et al. 2016). The following antibodies: Fibronectin, Laminin, CollagenI, CollagenIV, and V5 were validated by Western blot according to their correct molecular weight listed on the antibody specifications. CK7, pHH3, Cleaved caspase-3 were validated by immunohistochemistry. YAP antibody was validated in (Bessonard, S. et al. 2015). The antibody we raised against an ADAMTS18 peptide was validated by Western blot shown Supplementary Figure 4. It did not work in our hands for mouse samples as shown in the IHC of mouse mammary glands (Response to reviewer 1). ADAMTS18 IHC on human tissue (Figure 8) is in line with the RNAscope of Figure 1.

Eukaryotic cell lines

Policy information about cell lines

Cell line source(s)

MCF-7 cells were purchased from American Type Culture Collection (ATCC) and MCF-10A were kindly provided by S. Duss.

Authentication

None of the 2 cell lines were authenticated. Cells were solely used for antibody validation.

Mycoplasma contamination

We confirm that both cell lines were tested negative for mycoplasma.

Commonly misidentified lines
(See ICLAC register)

No commonly misidentified cell lines were used in this study.

Animals and other organisms

Policy information about studies involving animals; ARRIVE guidelines recommended for reporting animal research

Laboratory animals

Mus musculus
Adamts18-/- mice were maintained in the C57BL/6JOlaHsd background.
129SV/C57BL6, mT/mG60, and NOD.Cg-Prkdcscid Il2rgtm1Wjl/SzJ (NSG) mice were purchased from Jackson Laboratories.
C57BL/6JOlaHsd mice purchased from Harlan Laboratories.
Col18a1+/- 61, MMTV::Cre (lineA) , Wnt4+/- , Wnt4fl/fl, and Tg-Act-EGFP mice were maintained in the C57BL/6JOlaHsd background. Female pubertal mice used in this study are 5 to 6 weeks old, whereas female adult mice were 14-25 weeks old.

Wild animals

No wild animals were used in this study

Field-collected samples

No field-collected samples were used in this study

Ethics oversight

All mice were maintained and handled according to Swiss guidelines for animal safety and experiments were performed in accordance with protocols approved by the Service de la Consommation et des Affaires Vétérinaires of Canton de Vaud, Switzerland. The study was approved by the Commission cantonale d'éthique de la recherche sur l'être humain (CER-VD 183/10).

Note that full information on the approval of the study protocol must also be provided in the manuscript.

Human research participants

Policy information about studies involving human research participants

Population characteristics	Breast tissue was obtained from women undergoing reduction mammoplasties with no previous history of breast cancer, who gave informed consent. Information about the age, reproductive history, hormone levels, .. etc are stored in our Slims electronic-notebook (ELN) and are available upon request.
Recruitment	Samples were examined by the pathologist to be free of malignancy and processed as previously described (G. Sflomos, M. Shamseddin, C. Brisken, An Ex vivo Model to Study Hormone Action in the Human Breast, , 1–6 (2015)).
Ethics oversight	The cantonal ethics committee approved the study (183/10).

Note that full information on the approval of the study protocol must also be provided in the manuscript.

Flow Cytometry

Plots

Confirm that:

- [x] The axis labels state the marker and fluorochrome used (e.g. CD4-FITC).
- [x] The axis scales are clearly visible. Include numbers along axes only for bottom left plot of group (a 'group' is an analysis of identical markers).
- [x] All plots are contour plots with outliers or pseudocolor plots.
- [x] A numerical value for number of cells or percentage (with statistics) is provided.

Methodology

Sample preparation	Mammary glands from adult mice (15-24 weeks old) were harvested from adult mice, mechanically and enzymatically digested using parallel razor blades, and a cocktail of 3mg/ml Collagenase A, 100U/ml Hyalurodinase in DMEM/F12 for 1.5-2 hours on a 40 RPM shaker at 37 degrees Celsius. Cell clusters were then further dissociated with 0.025% Trypsin/EDTA, 5 mg/ml Dispase II, and 5 mg/ml DNase I, resuspended in red blood cell lysis buffer for 2-3 minutes, and filtered through a 40 um cell strainer. Cells were then labeled using anti-CD24-PE-Cy7 (cat# 560536, BD Pharmingen), anti-CD49f-APC (cat# 313616, Biolegend), anti-CD31-BV421 (cat# 563356, BD Pharmingen), anti-CD45-BV421 (cat# 563890, BD Pharmingen) and anti-Ter119-BV421 (cat# 563998, BD Pharmingen), and analyzed by flow cytometry.
Instrument	FACSAria (Becton Dickinson)
Software	FlowJo (version 9.8.2; https://www.flowjo.com) was used to analyze the distribution of labeled cells post-sorting.
Cell population abundance	Total cell number for Adamts18 WT was on average 8 million cells, as for the KO, the average of total cell number analyzed was 5.36 million cells (figure 4b). After lineage depletion, the percentage of stromal, luminal, and myoepithelial cells were on average 45, 23, and 28% of the total cell population analyzed.
Gating strategy	Living cells were selected by forward scatter, side scatter, doublets discrimination and by DAPI dye exclusion. Luminal, myoepithelial, and stromal cells were selected based on the expression of CD24 and CD49 markers and exclusion of CD31, CD45, and Ter119.

- [x] Tick this box to confirm that a figure exemplifying the gating strategy is provided in the Supplementary Information.
